# Effects of Low-Dose Microwave on Healing of Fractures with Titanium Alloy Internal Fixation: An Experimental Study in a Rabbit Model

**DOI:** 10.1371/journal.pone.0075756

**Published:** 2013-09-26

**Authors:** Dongmei Ye, Yiming Xu, Han Zhang, Tengfei Fu, Lan Jiang, Yuehong Bai

**Affiliations:** Department of Rehabilitation, Shanghai Sixth People’s Hospital, Shanghai Jiao Tong University, Shanghai, China; Delft University of Technology (TUDelft), Netherlands

## Abstract

**Background:**

Microwave is a method for improving fracture repair. However, one of the contraindications for microwave treatment listed in the literature is surgically implanted metal plates in the treatment field. The reason is that the reflection of electromagnetic waves and the eddy current stimulated by microwave would increase the temperature of magnetic implants and cause heat damage in tissues. Comparing with traditional medical stainless steel, titanium alloy is a kind of medical implants with low magnetic permeability and electric conductivity. But the effects of microwave treatment on fracture with titanium alloy internal fixation *in* *vivo* were not reported. The aim of this article was to evaluate the security and effects of microwave on healing of a fracture with titanium alloy internal fixation.

**Methods:**

Titanium alloy internal fixation systems were implanted in New Zealand rabbits with a 3.0 mm bone defect in the middle of femur. We applied a 30-day microwave treatment (2,450MHz, 25W, 10 min per day) to the fracture 3 days after operation. Temperature changes of muscle tissues around implants were measured during the irradiation. Normalized radiographic density of the fracture gap was measured on the 10th day and 30th day of the microwave treatment. All of the animals were killed after 10 and 30 days microwave treatment with histologic and histomorphometric examinations performed on the harvested tissues.

**Findings:**

The temperatures did not increase significantly in animals with titanium alloy implants. The security of microwave treatment was also supported by histology of muscles, nerve and bone around the implants. Radiographic assessment, histologic and histomorphometric examinations revealed significant improvement in the healing bone.

**Conclusion:**

Our results suggest that, in the healing of fracture with titanium alloy internal fixation, a low dose of microwave treatment may be a promising method.

## Introduction

Internal fixation of broken bones was considered to be the most prevalent methods of fracture treatment [1]. However, a part of patients suffered from painful symptoms such as implant-related pain, delayed union or nonunion so forth after the surgery [2]. Typically, clinical hyperthermia induced by focused microwave has been applied to patients with muscle-skeletal injuries [3-7]. The physiological effects of microwave are those of heat in deep the body generally. These include increased temperature over 40°C [8], increased blood flow [9,10], decreased pain [11], and alterations in the physical properties of fibrous tissues [12,13]. The clinic application of microwave can accelerate the resolution of haematoma and fracture healing, and increase range of movements of a joint by correcting contractures and decreasing viscosity of body fluids [3]. According to its effects, microwave treatment is clinically applied by doctors and therapist to the cure and rehabilitation in injuries bone [13-18]. However, a contraindication for microwave extensively documented in the literature is surgically implanted metal plates, screws, and pins in the treatment field [14]. As a kind of electromagnetic wave, microwave irradiation can be reflected, refracted or transmitted at boundaries [14,19]. Additionally, the eddy current stimulated by electromagnetic field could cause Joule heating of implants as well. All these factors would result in rapid temperature rise, even heat damages in local tissues [20,21]. The previous studies *in vitro* found that the temperature increase of implanted metallic plates might cause tissues ambustion in a frequency field of 900 MHz [22] and 27 MHz [23]. However, some doctors believed that this recommendation of contraindication appeared to be based on "common sense" and consensus rather than evidence-based practice *in vivo*. The findings of researches *in vitro* on radiofrequency (RF) electromagnetic field indicated that the presence of implantations should seldom be a risk, though 1800 [24] and 2450MHz [25,26] microwave irradiation could enhance the values of specific absorption rate (SAR). Besides, Seiger [27] and Draper [28] improved ankle range of motion (ROM) for patients with titanium alloy metal implants by pulsed shortwave diathermy. No discomfort, pain, or burning sensation was complained meanwhile. With regard to patient’s safety, invasive temperature monitoring was not applied during the microwave treatment in the two clinic studies mentioned above. However, doctors and physical therapists were more concerned with the efficiency and safety of microwave exposure in patients who were implanted titanium and its alloys since such kind of implants are widely used in clinic.

The current study was based on the hypothesis that, due to its physical characteristics [29], a titanium alloy dose not disrupt the uniformity of the field in an electromagnetic field and cause no heat accumulation under a proper dose of microwave irradiation. Accordingly, we applied a low-dose microwave treatment on the fracture models of rabbit femurs with internal fixation titanium alloy. The security of the treatment was assessed by temperature measurement and histodiagnosis of the tissues adjacent to the implants. Moreover, to assess whether such a dose of microwave therapy might accelerate the fracture healing, radiographic assessment, histomorphometery and histologic grade of callus were investigated. The preclinical research we did was to provide evidence for microwave treatment on fractures with titanium alloy internal fixation devices.

## Materials and Methods

### Ethics statement

All the experimental procedures involving animals were conducted under a protocol reviewed and approved by the Animal Welfare and Ethics Committee of Shanghai Sixth People’s Hospital (Permit Number: SYXK(沪) 2011-0128). All animal work was carried out in accordance with national and international guidelines to minimize suffering to animals.

### Animal, treatment and grouping

Thirty-eight (18 females and 20 males) New Zealand healthy adult white rabbits weighing from 2.0 to 3.2 kilograms (an average of 2.5 kilograms) were used in this experiment. The animals were fed in Laboratory Animal Center at Shanghai sixth people’s hospital, and were housed in a temperature, humidity, and light controlled environment.

After one week feeding adaptation, the animals were accurately weighed and randomly divided into three groups. Both the microwave treatment group (n=19) and implanted control group (n=16) were operatively implanted with a titanium alloy implants on bone fractures, while only the microwave treatment group received microwave treatment after the surgery. The non-implanted control group (n=3) was of bone lesions without titanium alloy implants, which was only used in the temperature measurement study (Table 1).

**Table 1:** Experimental Design and the Number of Animals Used in Each Test.

	Non-implanted Control Group (n=3)	Implanted Control Group (n=16)	Microwave Treatment Group (n=19)
Microwave Treatment	0	0	16
Temperature Measurements	3	0	3*
Radiographic Assessment	0	10	10
Histomorphometery	0	8	8
Histologic Grading	0	13	14

* These animals were selected randomly from the microwave treatment group after the surgery and received no microwave irradiation before the temperature measurement.

### Animal model construction

**Figure 1:**
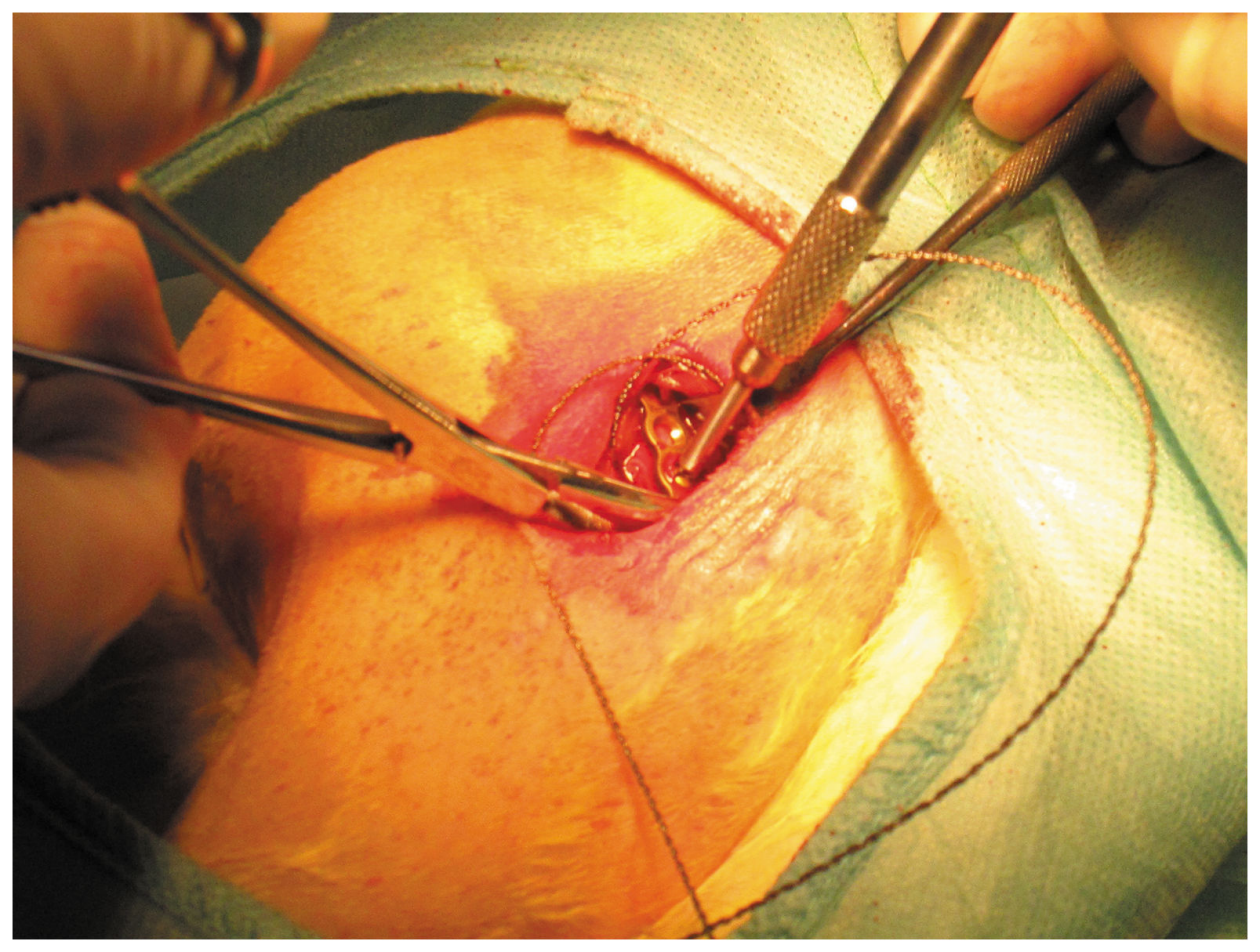
Intraoperative photographs. The miniscrew holes were prepared and an osteotomy was made by a 3mm fissured burr. The fractured segments were fixed with a miniplate and miniscrews of titanium alloy.

The rabbits were anesthetized with an intravenous injection of pentobarbital sodium (30 mg/kg). The femur was exposed via longitudinal skin incision on the outside of right hind limb. Transverse osteotomy with a 3.0 mm bone defect was created in the middle of femur. The internal fixation system (LCP, T-plate 2.4, Synthes Company, USA) was composed of a titanium alloy plate (4.60 ± 0.24 X 0.42 ± 0.08cm) and screws, which were planted on the right femoral upper end of the implanted control group and microwave treatment group (Figure 1). To act as an operated control, the non-implanted control animals underwent the same surgical procedure without titanium alloy implants. All rabbits were injected intramuscularly with penicillin (800,000 units) once per day during the 3 days following surgery.

### Microwave treatment

Three days after operation, microwave therapy was applied on the treatment group. The treatment regimen consisted of the right upper thigh. The applicator (RM-170A, ITO Company, Japan) was connected to a 2450 MHz microwave generator (PM-800, ITO Company, Japan) with power output ranging from 0-200W. A 25W continuous-wave microwave exposure lasted for 10 min per day [15,30]. The non-contact applicator was perpendicular to the lesion and 10 centimeters away from the skin. Similar procedure was repeated for the implanted control group with the microwave apparatus in the off position. In the non-implanted control groups, the legs were observed without further microwave treatment. In order to exclude the influence of body temperature changes and other factors in one day, we provided all the microwave treatment at the same time of the day.

### Temperature measurements

In all, 3 animals from the microwave treatment group and 3 animals from the non-implanted control group were used in this experiment. Anti-interference couple thermometer (FHC, ME-04008, BOWDOINHAM, USA), which is not influenced by the magnetic field and the radio frequency irradiation, was used for temperature measurements. The thermal probes of 8 centimeters length were placed into two sites for temperature measurements, including deep muscles (5mm above the middle hole of the titanium alloy plate) and superficial muscles (15mm above the hole). For placement in muscle, the vastus lateralis and the biceps femoris muscles were separated by blunt dissection, and the probes were placed with the recording side facing biceps femoris. The temperatures of non-implanted control group were tested in the same positions of the treatment regimen as well. The temperature was recorded every minute for 15 minutes while the microwave treatment was taking. The temperature of the laboratory was maintained at 24°C.

### Radiographic assessment

The healing of fracture was assessed by anteroposterior plain radiographs. The same X-ray machine and settings were used for all radiographs just after the operation and on the 10th and 30th day of the microwave treatment. Also, the same light source was used for the optical densitometry. An optical densitometer was used to measure radiographic density of the radiographic films. However, there may be variations within radiographs with respect to film processing. A standard aluminum step wedge was used to calibrate the optical densitometer and check for its reproducibility. Radiographs of the wedge were taken 10 times, and the film’s densities at each layer were measured by the optical densitometer (Table 2).

**Table 2:** Film Densities of 10 Radiographs Taken from a Standard Aluminum Step Wedge Measured by the Optical Densitometer at Different Layers of the Wedge.

	Lay Thickness (mm)										
Trial No.	0	1	2	3	4	5	6	7	8	9	10
1	1.71	1.35	1.19	1.04	0.99	0.81	0.74	0.66	0.60	0.58	0.52
2	1.71	1.37	1.16	1.01	0.89	0.80	0.72	0.64	0.61	0.57	0.54
3	1.69	1.33	1.14	1.00	0.88	0.78	0.71	0.67	0.60	0.56	0.55
4	1.67	1.33	1.18	1.03	0.90	0.82	0.73	0.67	0.59	0.55	0.51
5	1.65	1.34	1.19	1.03	0.91	0.82	0.73	0.66	0.62	0.56	0.52
6	1.69	1.35	1.18	1.04	0.91	0.81	0.72	0.65	0.60	0.55	0.52
7	1.67	1.37	1.16	1.01	0.88	0.79	0.71	0.65	0.60	0.56	0.52
8	1.68	1.34	1.18	1.03	0.91	0.79	0.71	0.64	0.59	0.55	0.53
9	1.71	1.35	1.14	1.00	0.88	0.82	0.72	0.65	0.60	0.56	0.52
10	1.67	1.36	1.17	1.02	0.89	0.79	0.71	0.68	0.63	0.56	0.55
Mean	1.69	1.35	1.17	1.02	0.90	0.80	0.72	0.66	0.60	0.56	0.53
SD	0.02	0.01	0.02	0.02	0.03	0.02	0.01	0.01	0.01	0.01	0.01

### Histology and Histomorphometery

After 10 days of microwave treatment, four rabbits from each group were sacrificed and eight intact samples were obtained for histologic examination. The remaining animals were killed at the end of 30-day treatments. Four intact samples from each group in addition to 5 samples from the implanted control group and 6 samples from the microwave treatment group were included for the same examination. The specimens were fixed in 10% formalin and decalcified with 10% nitric acid. After being embedded in paraffin, 5µm-thick longitudinal sections were prepared and stained with hematoxylin and eosin or Masson’s trichrome. Two slides from each specimen, one stained with hematoxylin-eosin and the other stained with trichrome, were examined. A panel of pathologists (LZW,JQ, DJX) who were blinded to the treatment allocation scored all the stained sections according to mineralization amounts of the fracture gap, using a grading system described by Perry et al [31], in which grade 1 indicated fibrous union, grade 2 indicated predominantly fibrous tissue with some cartilage, grade 3 indicated equal amounts of fibrous tissue and cartilage, grade 4 indicated all cartilage, grade 5 indicated predominantly cartilage with some woven bone, grade 6 indicated equal amounts of cartilage and woven bone, grade 7 indicated predominantly woven bone with some cartilage, grade 8 indicated woven bone, grade 9 indicated woven bone and some lamellar bone, and grade 10 indicated lamellar bone. The mean score was calculated for each group.

For histomorphometric evaluations of bone, undecalcified sections of 4 intact samples from each group were prepared after 10 and 30 days of microwave treatment. The specimens were fixed in 10% buffered formalin, dehydrated in increasing concentrations of ethanol, from 70% to 99% during 12 days, and embedded in methylmethacrylate. The 50-µm-thick sagittal sections were prepared using an electric diamond saw and grinding system, and stained with toluidine blue. Digital images of the sections were obtained by a digital camera attached to an microscope at a magnification rate of ×25. The images were transferred to a personal computer, and measurements at the fracture gap were made by histomorphometry software (simple PCI system; Hamamatsu, Sewickley, PA, USA). The fracture gap was selected for examination. The nomenclature and calculations for bone histomorphometry were applied in accordance with the report of the American Society for Bone and Mineral Research [32].

Muscular tissue and nervous tissue around implants were removed and fixed in 10% formalin at the end of the scheduled. The 5 µm sections were prepared and stained with haematoxylin and eosin following standard techniques for microscopic examination. For transmission electron microscopy, skeletal muscle tissue and nervous tissue were fixed with 2.5% glutaraldehyde for 16 h at 4°C, postfixed with 2% OsO_4_ for 2 h at 4°C, dehydrated by an ascending ethanol series, passed through propylene oxide, and then embedded in resin. Ultrathin sections (90 nm thick) on mesh grids were stained with uranyl acetate and lead acetate and examined with an electron microscope.

### Statistic

Statistical analyses were performed with SPSS 19.0 for Windows (SPSS, Chicago, USA). All the results are presented as the mean value ± standard deviation. Differences between groups were tested using t-test or analysis of variance (ANOVA). For all analyses, *P* values were two-tailed, and *P*<0.05 was considered statistically significant.

## Results

### Microwave Treatment Caused No Significant Heated Damage In Tissues Around The Implants

During the microwave irradiation, temperatures were measured in deep and superficial muscle tissues meanwhile. Temperature changes were show in table 3. No difference was found between non-implanted control group and microwave treatment group in peak temperatures of the deep muscles (39.73 ± 0.23°C vs. 39.83 ± 0.25, *P*=0.639) and superficial muscles (39.90 ± 0.10°C vs. 39.67 ± 0.21°C, *P*=0.155). Although the temperature changes (T_peak_-T_vally_) of microwave treatment group in deep muscles were higher than those of non-implanted control group (3.57 ± 0.44 and 3.50 ± 0.36 respectively), no statistic difference was observed between the two groups (*P* =0.82). So was the temperature increasing in the superficial muscles (control group vs. treatment group: 3.77 ± 0.36 and 3.60 ± 0.41, *P* =0.28).

**Table 3:** The Temperature Rises in Deep Muscles and Superficial Muscles during Microwave Irradiation.

	Deep Muscles (Mean ± SD)			Superficial Muscles(Mean ± SD)		
Time	Implanted Control Group	Microwave Treatment Group	*P* †	Implanted Control Group	Microwave Treatment Group	*P* †
0	36.23±0.06	36.27±0.23	0.820	36.13±0.15	36.07±0.06	0.519
1	36.87±0.42	36.97±0.25	0.740	36.67±0.25	36.63±0.25	0.879
2	37.57±0.25	37.37±0.21	0.349	37.33±0.42	36.90±0.10	0.154
3	37.67±0.25	37.70±0.26	0.882	37.57±0.31	37.10±0.17	0.083
4	37.93±0.21	38.10±0.20	0.374	37.80±0.36	37.23±0.15	0.066
5	38.17±0.23	38.40±0.30	0.346	38.07±0.25	37.47±0.29	0.053
6	38.50±0.20	38.73±0.15	0.184	38.37±0.32	37.67±0.29	0.049
7	38.73±0.21	38.93±0.12	0.219	38.63±0.29	37.90±0.35	0.048
8	39.00±0.26	39.03±0.12	0.851	38.87±0.35	38.13±0.32	0.056
9	39.10±0.17	39.23±0.06	0.275	39.10±0.17	38.40±0.44	0.061
10	39.30±0.17	39.43±0.06	0.275	39.30±0.17	38.60±0.44	0.061
11	39.47±0.15	39.60±0.10	0.275	39.53±0.12	38.87±0.47	0.077
12	39.60±0.20	39.70±0.10	0.482	39.67±0.15	39.00±0.46	0.075
13	39.70±0.20	39.73±0.15	0.830	39.77±0.12	39.20±0.44	0.095
14	39.70±0.20	39.83±0.15	0.411	39.87±0.06	39.40±0.46	0.155
15	39.73±0.23	39.83±0.25	0.639	39.90±0.10	39.67±0.21	0.155

Data were expressed as mean ± SD.

† Differences were assessed by performing t-test.

**Figure 2:**
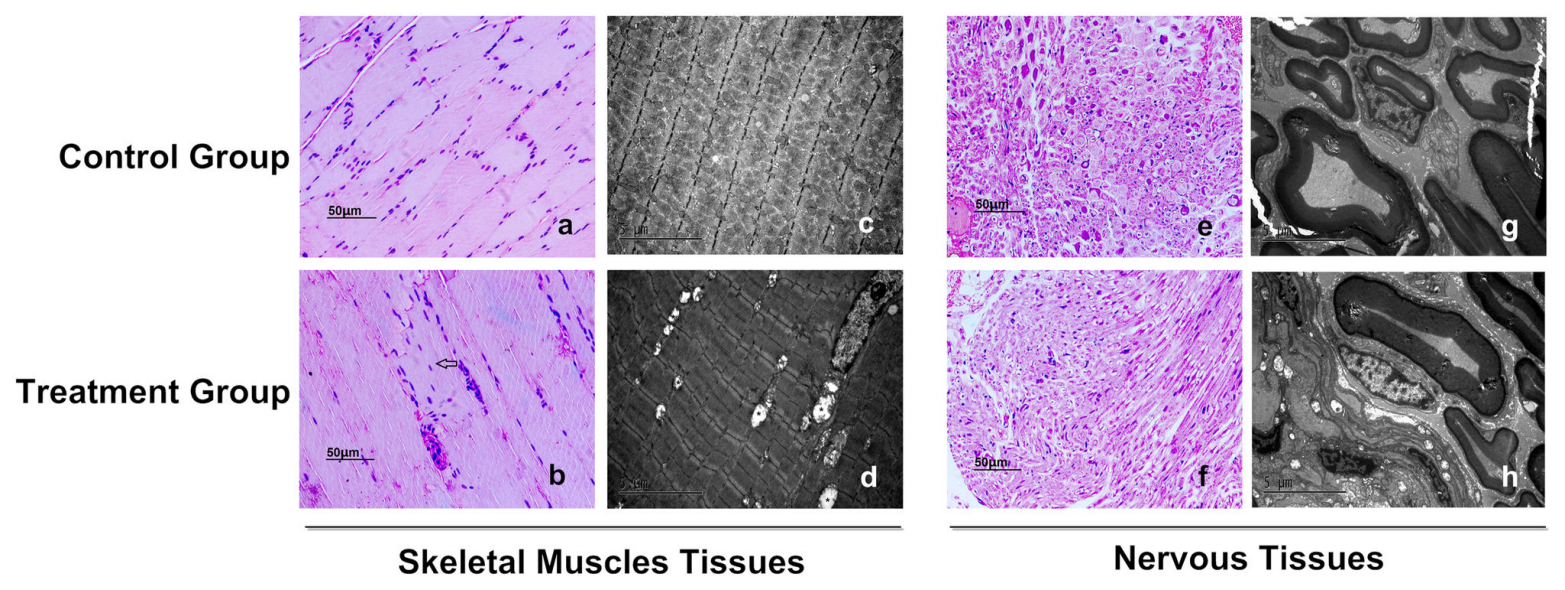
Histological analysis of thigh muscle and nervous tissues adjacent to implants. a, b Sections of thigh muscle from implanted control group (a) and microwave treatment group (b) were stained with hematoxylin and eosin after 30-day microwave treatment. Arrow shows swelling myocytes observed occasionally in the treatment group. c, d Transmission electron microscopy of skeletal muscle from the implanted control group (c) and microwave treatment group (d). Asterisk shows abnormal mitochondria and mitochondrial cristae decreasing in the treatment group. e-h The optical photomicrographs (e, f) and transmission electron microscopy photographs (g, h) of nervous tissues adjacent to implants. No abnormal morphological changes are investigated in either control or treatment group. Scale bars: 50µm (a, b, e and f); 5µm (c, d, g and h).

We next investigated whether a long-term course of the on titanium implanted subjects could cause heat damage to the tissues around the implants. After 30-day microwave treatment, skeleton muscle tissues adjacent to the titanium alloy were investigated histologically. The muscles from the treatment and control group were stained with hematoxylin and eosin (Figure 2 a, b). For the treatment group, most myocytes displayed normal morphology though swelling myocytes were observed occasionally in the treatment field. We further performed transmission electron microscopic analysis of the skeletal muscle. The muscle of the treatment group showed mitochondrial swelling and mitochondrial cristae loss, which were not observed in control group (Figure 2 c, d). The nerve tissues adjacent to the implants were observed with both light microscope and transmission electronic microscope. No abnormal morphological changes were investigated in either treatment or control group (Figure 2 e-h).

### Microwave Treatment Accelerated Fracture Healing

**Figure 3:**
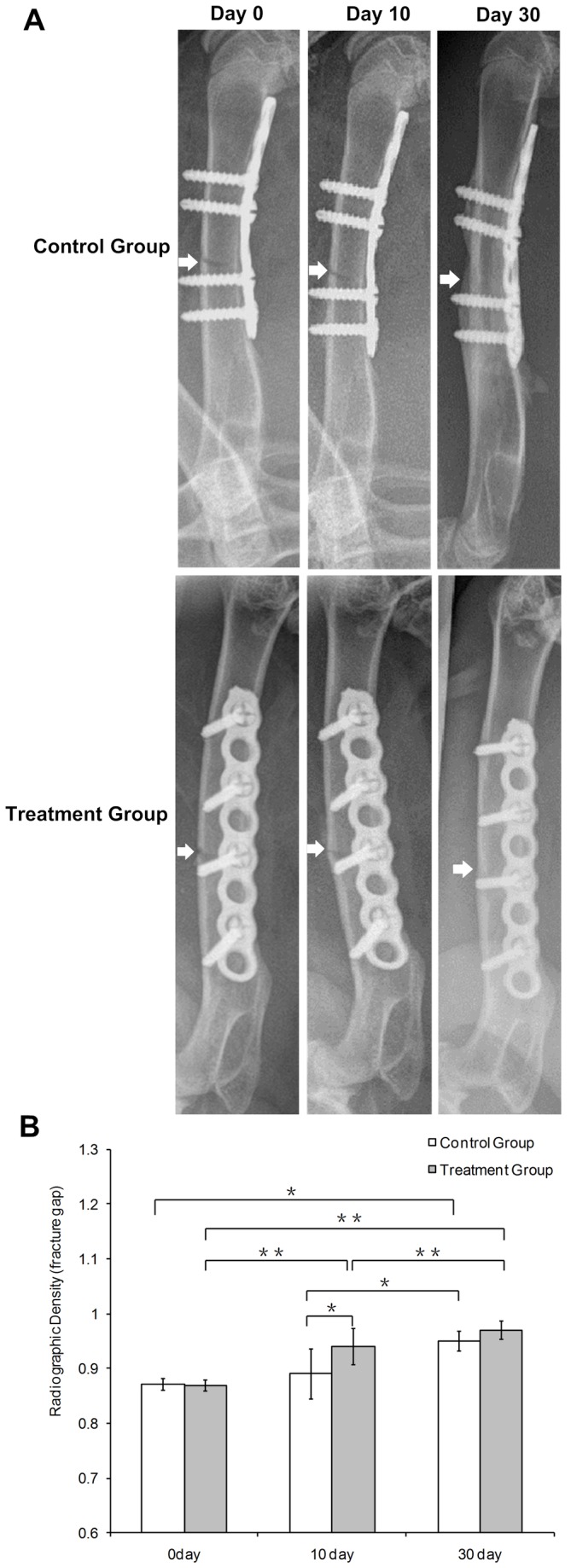
Radiographs of rabbit femur and the radiographs analysis. (A) Radiographs of rabbit femur were right after the surgery and on the 10th and 30th day of microwave treatment. Arrows show the fracture gaps. Fractures generally heal well after 30-day microwave treatment, and no obvious fracture gap was found in both groups. (B) Normalized radiographic density of femur in implanted control and microwave treatment groups after the surgery and on the 10th and 30th day of microwave treatment. Data represent mean ± SD. Differences were assessed by performing t-test. *P <0.05, **P <0.01.

Radiographic examinations showed that all injured femora were in the process of normal bone healing. Callus formation was seen in the control group as well as treatment group. But it was more evident in the microwave treatment group compared with the implanted control group, indicating a positive effect of microwave treatment (Figure 3A). Within-group statistical analysis showed that, within the implanted control group and within the treatment group, this measure increased as time point increased (control group: *P*=0.001; treatment group: *P*<0.001; Figure 3B). Differences between implanted control and treatment groups were significant on the 10th day of the microwave treatment (*P*=0.03; Figure 3B). However, normalized radiographic density was not significantly different on the 30th day in the treatment group compared with that in the control group (*P*=0.12; Figure 3B).

**Figure 4:**
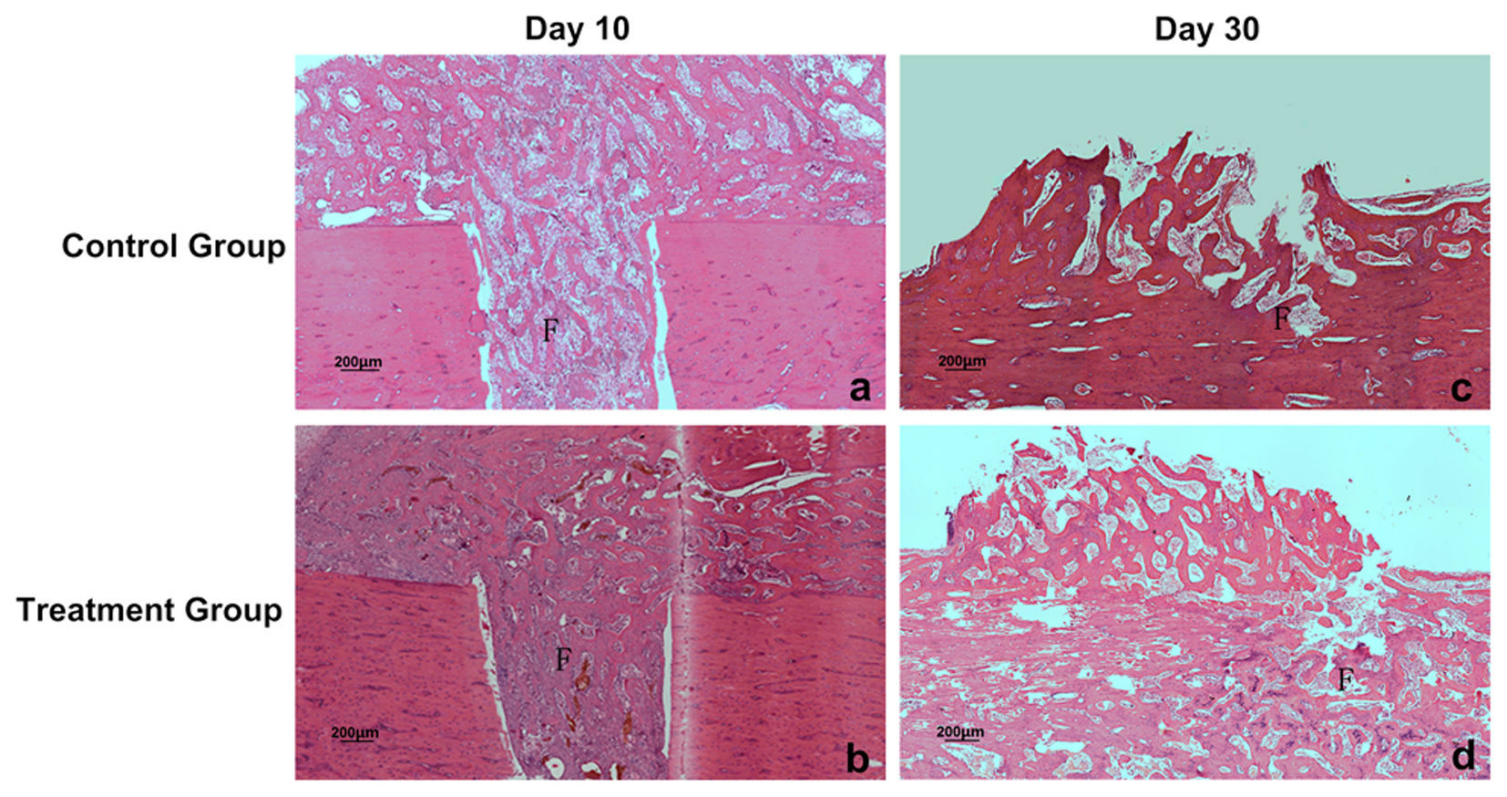
Histological evaluation of bone healing. a-d, After 10 and 30 days microwave treatment, sections of fracture gap from implanted control group (a, c) and microwave treatment group (b, d) were stained with hematoxylin and eosin for histological evaluation. F: fracture gap. Scale bars: 200µm.

Histological changes of bone were studied microscopically. There was no evidence of ambustion in osseous tissues after 30-day microwave treatment (Figure S1). The histologic grade of callus in fracture gap was studied microscopically at two time points. At day 10, the mean histological grade was 2.75 ± 0.50 for the microwave treated group and 1.75 ± 0.50 for the implanted control group. There was a significant difference between the two groups (*P*=0.03). At day 30, the mean histologic grade was 7.08 ± 0.52 for the microwave treated group compared with 6.45 ± 0.69 for the implanted control group (Figure 4 and Table 4). The difference between the two groups was significant (*P*=0.025).

**Table 4:** Comparison of the Histologic and Histomorphometric Data.

Parameter	Implanted Control Group		Microwave Treatment Group	
	10 day	30 day	10 day	30 day
Histologic Grading	1.75±0.50	6.45±0.69	2.75±0.50*	7.08±0.52*
Bone Volume^★^ (%)	17.15±3.27	56.23±7.12	22.75±2.70**	66.09±3.09*
Trabecular Thickness^▲^ (µm)	5.27±1.10	20.73±3.12	7.13±1.69*	23.27±2.01
Trabecular Separation^†^ (µm)	26.36±8.23	17.13±6.16	24.93±8.71	11.96±1.64
Node-terminus Ratio^‡^ (/mm)	2.08±0.56	4.85±1.83	2.64±0.32*	7.20±1.91*

★ Bone volume (BV/TV) Ratio of mineralized and unmineralized bone volume to the total tissue volume estimated from the analyzed section.

▲ Trabecular thickness (Tb.Th) Trabecular width/1.2 (1.2 is a correction factor for section obliquity).

† Trabecular separation (Tb. Sp) Trabecular thickness × ([100/BV/TV] - 1). ‡ Node-terminus ratio (NNd/NTm) Ratio of node (a point at which 3 or more struts are joined) number to terminus (a point at which the trabecula is not joined to any other trabecula) number. A skeletonized trabecula is called a strut. * *P*<0.05, **P<0.01 vs. control group of the same time point

The histomorphometric data regarding the mean values of bone volume, trabecular thickness, trabecular separation, and node-terminus ratio were given in Table 4. Bone volume and node-terminus ratio were significantly different between implanted control and treatment groups after 10-day and 30-day microwave treatment (all P<0.05). Additionally, significant difference in trabecular thickness were also observed between the two groups after 10-day microwave treatment (*P*=0.048)

## Discussion

Metallic implants in treated regions are described as a contraindication for RF treatment because of the intense and highly heating occurrence. Two mechanisms have been proposed for such an undesirable heating. The reflection of electromagnetic wave from metal surfaces was thought to be one reason [33]. Double dose of RF irradiation would heat the tissues adjacent to the metal implants. Comparing with osseous tissue, muscle tissues are of high water content which characteristically absorbs microwaves strongly because that microwave heats water preferentially [34]. Therefore, the present study was concerned with the temperature changes of muscles adjacent to the metal implants. Additionally, the eddy current stimulated by electromagnetic field is the other reason. The difference of eddy current intensity derived from the physical properties of the materials. Comparing with traditional medical stainless steel, titanium alloy is a kind of medical implants with low magnetic permeability and electric conductivity. For these reasons, lower RF heating was observed at the border of titanium alloy implants and phantom *in vitro* [35,36]. Implants made by titanium alloy are non-ferromagnetic and produce much less artifacts than ferromagnetic implants made of stainless steel at magnetic resonance (MR) imaging [37-39]. RF heating of cobalt-chromium alloy implants and titanium alloy implants was evaluated *in vitro* by Muranaka. The maximum temperature rise for titanium implant was obviously lower than that for cobalt-chromium [35,40]. Hence, the current study was based on the assumption that there is no dramatic temperature raise in tissues around titanium alloy implants resulting from a low dose microwave exposure. The findings of our research showed that such a dose of microwave irradiation did not dramatically increase the temperatures in the skeletal muscle tissues around titanium alloy.

In the current research, the results show that the temperature increase of muscles adjacent to titanium alloy implants was lower than 40°C. A previous study found that enzyme and proteins in the cell began to degeneration when the body temperature goes over 43°C [41,42]. Cumulative equivalent minutes at 43°C (CEM_43_) are the accepted metric for thermal dose assessment that correlates well with thermal damage in a variety of tissues. The calculation of CEM_43_ involves knowledge of thermal history:

CEM_43_ = Δt ^*^ R ^43-T^

Where Δt signifies summation over the length of exposure, T is the average temperature during time interval t, and R is a constant equal to 0.25 for T < 43°C and 0.5 for T >43°C [43,44]. The histology of skeleton muscle in pig revealed that a minor damage appeared at 30 CEM_43_ [45]. The thermal dose scale was 240 CEM_43_ for leg muscle of rabbits and pigs, which corresponded to an irreversible lethal dose for cells [46-48]. In the current study, the thermal doses (CEM_43_) of microwave treatment group were 0.08 CEM_43_ in deep muscles and 0.03 CEM_43_ in superficial muscles (the data in table 3 analysed). However, we observed swelling myocyte occasionally though the peak temperatures were lower than 40°C in muscles. The reason might be the mitochondrial swelling and cristae loss, which prevented mitochondrial ATP/ADP exchange [49]. It was noteworthy that the sign of mitochondrial swelling was typical features of mitochondrial stress. The previous studies found that the mitochondria could change their size in a phasic manner, swelling when cells were exposed to the temperature of 40°C, and all these shape changes were reversible when the temperature returns to normal [50,51]. Thus we can conclude that such a dose of microwave treatment on the subject with titanium alloy can’t cause irreversible damages to the muscle around the implants. However, burn injuries could happen with the increase of microwave dose. In our preliminary experiment, heat effects of different doses of microwave irradiation on the muscles adjacent to the titanium alloy implants were studied. We found that if the dose increased to 2,450MHz, 60W, the temperature of muscles around the implants would reach 43°C within 10min (data not shown). Moreover, microwave is relatively insensitive to high impedance and has a deeper penetration profile [52]. Since bone has low conductivity (high impedance), less Joule heat is caused by reflection of electromagnetic wave and electrical resistance compared with muscles. On the other hand, bone, especially cortical bone, has low heat-sink strength from tissue volume by blood perfusion. It means that a long duration of hyperthermia might induce heat damages in bones. Therefore, we adopted a shorter duration of microwave exposure mentioned by Chang [15]. The microscopical results of the current research provided confirmation that such a short-duration microwave treatment caused no heat damage in osseous tissue and bone marrow around the titanium alloy (Figure S1).

The results of our current studies examine the effects of microwave continuous irradiation on promoting femoral fracture healing in rabbit. Many technical obstacles, such as temperature control, burns, and the fact that microwaves from an external source penetrate only 3-4 cm, are some of the reasons that microwave is not yet a clinically accepted modality of treatment. However, the use of microwave for extra-corporeal heating of bone seems feasible. Using a dedicated microwave hyperthermia system, Leon et al. systematically examined the effect of microwave hyperthermia on bone both *in vitro* and *in vivo* [16,17]. They demonstrated that hyperthermia promoted bone deposition. The effect of microwave is mainly induced by the alternating electromagnetic fields, which activates the dipoles in the molecule of the material [16]. The collisions of the molecules cause generation of heat inside the object [53]. Due to the dose of microwave we used, temperate changes were not dramatic in the treatment field. Although this heating effect is small, some enzymes are exquisitely sensitive to small variations in temperature [54]. It was reported that 0.24 W/cm^2^ pulsed shortwave diathermy was safe for human with titanium implants [28,55], and 0.5 W/cm^2^ continuous microwave could produce thermal effect on fracture rehabilitation in rabbit [15,16]. In the present study, the effective treatment area of the applicator we used was 138.9 cm^2^ and the dose of continuous microwave was 0.18 W/cm^2^, which derived from a randomized clinical trial of microwave stimulation of bone healing we did previously. It was recognized as the minimum dose which has an effect on the femoral fracture patients without metal implants. In current study, we confirmed that the healing of fracture with titanium alloy implants could be accelerated by such low-dose of microwave treatment in animals. Although the dose we used was lower than that previous studies did, its security of femoral fracture fixed with titanium alloy plate was confirmed in rabbits. A favorable effect of microwave irradiation on bone union was also found, especially in the first 10 days treatment. The similar results were reported by Olchowik [56]. The results indicated that the low-dose of microwave therapy bring positive impacts in the early stage of fracture repair. But the mechanisms remained unclear. There seem to be three mechanisms involved. First of all, the most important physiological response induced by microwave is the regional increase in the blood flow [10]. Thus an increase in nutrients and oxygen in the treatment region would likely benefit to the bone healing. Additionally, microwave treatment has been advocated by several researchers to accelerate the resolution of haematoma which is diffusely observed near the fracture site in the early stage of bone trauma [9,57]. Moreover, at the molecular level, it is possible that microwave treatment accelerates or increases the release of local growth factors such as transforming growth factor β (TGF-β), fibroblast growth factor and platelet-derived growth factor – all of which have been reported to accelerate the earlier appearance and promote the proliferation of chondrocytes during the early phase of fracture healing [58,59].

A number of limitations of this study warrant mention. First, although there was no heat damage shown in histology, the temperature changes of bone should be observed in advanced researches because of the report that it could damage at 80 CEM43 [60]. Second, the small sample size of rabbits in each group, the limited number of specimens in temperature measurement and histomorphometery might not enable us to reach any firm conclusions. However, as all the results in this study were consistent with each other, we feel confident in our findings. Meanwhile, our further studies in the future with ample numbers of subjects and long-term follow-up may strengthen these findings and confirm the superiority of this treatment.

## Conclusion

Our study *in vivo* proved that the continuous-wave microwave treatment, at 2,450MHz, 25W, 10 minutes per day, did not dramatically increase the temperature in muscle tissues around titanium alloy implants. It did not cause irreversible heat damage in the tissues for a 30-day treatment as well. Additionally, such dose of microwave improved the femoral fracture healing in rabbit. Our results suggest that, in the healing of fracture with titanium alloy internal fixation, a low dose of microwave treatment may be a promising method.

## Supporting Information

Figure S1 **Histological manifestation of the treated femur adjacent to the screw after 30-day microwave treatment.** No morphologically discernible tissue injury was observed in cortical bone (a) or bone marrow (b) of the targeted bone segment. Asterisk: the location of implanted screw. Scale bars: 200µm.
(TIF)

## References

[1] Perren SM (2008) Fracture healing. The evolution of our understanding. Acta Chir Orthop Traumatol Cech 75: 241-246. PubMed: 18760078. 18760078

[2] Campbell R, Campbell D (2013) Imaging the Post-Operative Wrist and Hand. Imaging of the Hand and Wrist. Springer Verlag pp. 365-386.

[3] Giombini A, Giovannini V, Di Cesare A, Pacetti P, Ichinoseki-Sekine N et al. (2007) Hyperthermia induced by microwave diathermy in the management of muscle and tendon injuries. Br Med Bull 83: 379-396. doi:10.1093/bmb/ldm020. PubMed: 17942453. 17942453 10.1093/bmb/ldm020

[4] Pope G, Mockett S, Wright J (1995) A survey of electrotherapeutic modalities: ownership and use in the NHS in England. Physiotherapy 81: 82-91. doi:10.1016/S0031-9406(05)67050-2.

[5] Rabini A, Piazzini DB, Tancredi G, Foti C, Milano G et al. (2012) Deep heating therapy via microwave diathermy relieves pain and improves physical function in patients with knee osteoarthritis: a double-blind randomized clinical trial. Eur J Phys Rehabil Med 48: 549-559. PubMed: 22820824. 22820824

[6] Giombini A, Di Cesare A, Safran MR, Ciatti R, Maffulli N (2006) Short-term Effectiveness of Hyperthermia for Supraspinatus Tendinopathy in Athletes A Short-term Randomized Controlled Study. Am J Sports Med 34: 1247-1253. doi:10.1177/0363546506287827. PubMed: 16636345. 16636345 10.1177/0363546506287827

[7] Giombini A, Casciello G, Di Cesare MC, Di Cesare A, Dragoni S et al. (2001) A controlled study on the effects of hyperthermia at 434 MHz and conventional ultrasound upon muscle injuries in sport. J Sports Med Phys Fit 41: 521-527. PubMed: 11687773. 11687773

[8] Dewhirst MW, Viglianti BL, Lora-Michiels M, Hanson M, PJ H (2003) Basic principles of thermal dosimetry and thermal thresholds for tissue damage from hyperthermia. Int J Hyperthermia 19: 267-294 10.1080/0265673031000119006 12745972

[9] Wyper DJ, McNiven DR (1976) The effect of microwave therapy upon muscle blood flow in man. Br J Sports Med 10: 19-21. doi:10.1136/bjsm.10.1.19. PubMed: 963368. 963368 10.1136/bjsm.10.1.19 PMC1859362

[10] Sekins KM, Lehmann JF, Esselman P, Dundore D, Emery AF et al. (1984) Local muscle blood flow and temperature responses to 915MHz diathermy as simultaneously measured and numerically predicted. Arch Phys Med Rehabil 65: 1-7. PubMed: 6691788. 6691788

[11] Yatvin MB (1977) The influence of membrane lipid composition and procaine on hyperthermic death of cells. Int J Radiat Biol Relat Stud Phys Chem Med 32: 513-521. doi:10.1080/09553007714551301. PubMed: 338522. 338522 10.1080/09553007714551301

[12] Lehmann JF, Guy AW, Stonebridge JB, Warren CG, DeLateur BJ (1975) Temperature distribution produced in models by three microwave applicators at 433.92 megahertz. Arch Phys Med Rehabil 56: 145-151. PubMed: 1119923. 1119923

[13] DeLateur BJ, Stonebridge JB, Lehmann JF (1978) Fibrous muscular contractures: treatment with a new direct contact microwave applicator operating at 915 MHz. Arch Phys Med Rehabil 59: 488-499. PubMed: 718413. 718413

[14] Goats GC (1990) Microwave diathermy. Br J Sports Med 24: 212-218. doi:10.1136/bjsm.24.4.212. PubMed: 2097017. 2097017 10.1136/bjsm.24.4.212 PMC1478902

[15] Chang WH, Sun JS, Chang SP, Lin JC (2002) Study of thermal effects of ultrasound stimulation on fracture healing. Bioelectromagnetics 23: 256-263. doi:10.1002/bem.10009. PubMed: 11948604. 11948604 10.1002/bem.10009

[16] Leon SA, Asbell SO, Arastu HH, Edelstein G, Packel AJ et al. (1993) Effects of hyperthermia on bone. II. Heating of bone in vivo and stimulation of bone growth. Int J Hyperthermia 9: 77-87. doi:10.3109/02656739309061480. PubMed: 8433028. 8433028 10.3109/02656739309061480

[17] Leon SA, Asbell SO, Edelstein G, Arastu HH, Daskal I et al. (1993) Effects of hyperthermia on bone. I. Heating rate patterns induced by microwave irradiation in bone and muscle phantoms. Int J Hyperthermia 9: 69-75. doi:10.3109/02656739309061479. PubMed: 8433027. 8433027 10.3109/02656739309061479

[18] Lubner MG, Brace CL, Hinshaw JL, Lee FT Jr. (2010) Microwave tumor ablation: mechanism of action, clinical results, and devices. J Vasc Interv Radiol 21: S192-S203. doi:10.1016/j.jvir.2010.04.007. PubMed: 20656229. 20656229 10.1016/j.jvir.2010.04.007 PMC3065977

[19] Grant EH (1981) Biological effects of microwaves and radio waves. IEE Proc A Phys Sci Meas Instrum Manag Educ Rev 128: 602. doi:10.1049/ip-a-1.1981.0091.

[20] Shields N, Gormley J, O’Hare N (2002) Short-wave diathermy: current clinical and safety practices. Physiother Res Int 7: 191-202. doi:10.1002/pri.259. PubMed: 12528575. 12528575 10.1002/pri.259

[21] Guy AW (1987) Dosimetry associated with exposure to non-ionizing radiation: very low frequency to microwaves. Health Phys 53: 569-584. doi:10.1097/00004032-198712000-00001. PubMed: 3679822. 3679822 10.1097/00004032-198712000-00001

[22] Cooper J, Hombach V (1996) Increase in specific absorption rate in human heads arising from implantations. Electron Lett 32: 2217-2219. doi:10.1049/el:19961507.

[23] Ruggera PS, DM Wi tters, altzahn GvM, Bassen HI (2003) In vitro assessment of tissue heating near metallic medical implants by exposure to pulsed radio frequency diathermy. Phys Med Biol 48: 2919

[24] Virtanen H, Keshvari J, Lappalainen R (2007) The effect of authentic metallic implants on the SAR distribution of the head exposed to 900, 1800 and 2450 MHz dipole near field. Phys Med Biol 52: 1221-1236. doi:10.1088/0031-9155/52/5/001. PubMed: 17301450. 17301450 10.1088/0031-9155/52/5/001

[25] McIntosh RL, Anderson V, McKenzie RJ (2005) A numerical evaluation of SAR distribution and temperature changes around a metallic plate in the head of a RF exposed worker. Bioelectromagnetics 26: 377-388. doi:10.1002/bem.20112. PubMed: 15924346. 15924346 10.1002/bem.20112

[26] Virtanen H, Huttunen J, Toropainen A, Lappalainen R (2005) Interaction of mobile phones with superficial passive metallic implants. Phys Med Biol 50: 2689-2700. doi:10.1088/0031-9155/50/11/017. PubMed: 15901963. 15901963 10.1088/0031-9155/50/11/017

[27] Seiger C, Draper DO (2006) Use of pulsed shortwave diathermy and joint mobilization to increase ankle range of motion in the presence of surgical implanted metal: A case series. J Orthop Sports Phys Ther 36: 669-677. doi:10.2519/jospt.2006.2198. PubMed: 17017272. 17017272 10.2519/jospt.2006.2198

[28] Draper DO, Castel JC, Castel D (2004) Low-Watt Pulsed Shortwave Diathermy and Metal-Plate Fixation of the Elbow. Athl Ther Today 9: 28-32.

[29] Bernhardt JH (1992) Non-ionizing radiation safety: radiofrequency radiation, electric and magnetic fields. Phys Med Biol 37: 807-844. doi:10.1088/0031-9155/37/4/001. PubMed: 1589456. 1589456 10.1088/0031-9155/37/4/001

[30] Li J (2005) fracture of the extremities. In: Li J clinical guidelines - physical medicine and rehabilitation. Beijing, China: People’s Medical Publishing House p. 54.

[31] Perry AC, Prpa B, Rouse MS, Piper KE, Hanssen AD et al. (2003) Levofloxacin and trovafloxacin inhibition of experimental fracture-healing. Clin Orthop Relat Res: 95-100. PubMed: 12966282. 10.1097/01.blo.0000087322.60612.14 12966282

[32] Parfitt AM, Drezner MK, Glorieux FH, Kanis JA, Malluche H et al. (1987) Bone histomorphometry: standardization of nomenclature, symbols, and units. Report of the ASBMR Histomorphometry Nomenclature Committee. J Bone Miner Res 2: 595-610. PubMed: 3455637. 3455637 10.1002/jbmr.5650020617

[33] Skonieczki BD, Wells C, Wasser EJ, Dupuy DE (2011) Radiofrequency and microwave tumor ablation in patients with implanted cardiac devices: is it safe? Eur J Radiol 79: 343-346. doi:10.1016/j.ejrad.2010.04.004. PubMed: 20434862. 20434862 10.1016/j.ejrad.2010.04.004

[34] Akyol Y, Ulus Y, Durmus D, Canturk F, Bilgici A et al. (2012) Effectiveness of microwave diathermy on pain, functional capacity, muscle strength, quality of life, and depression in patients with subacromial impingement syndrome: a randomized placebo-controlled clinical study. Rheumatol Int 32: 3007-3016. doi:10.1007/s00296-011-2097-2. PubMed: 21898066. 21898066 10.1007/s00296-011-2097-2

[35] Muranaka H, Horiguchi T, Ueda Y, Usui S, Tanki N et al. (2010) Evaluation of RF heating on hip joint implant in phantom during MRI examinations. Nihon Hoshasen Gijutsu Gakkai Zasshi 66: 725-733. doi:10.6009/jjrt.66.725. PubMed: 20702992. 20702992 10.6009/jjrt.66.725

[36] Huidobro C, Larson B, Mynderse S, Myers JJ, Busel D et al. (2009) Characterizing Prostiva RF treatments of the prostate for BPH with gadolinium-enhanced MRI. ScientificWorldJournal 9: 10-16. doi:10.1100/tsw.2009.4. PubMed: 19151893. 19151893 10.1100/tsw.2009.4 PMC5823118

[37] Ganapathi M, Joseph G, Savage R, Jones AR, Timms B et al. (2002) MRI susceptibility artefacts related to scaphoid screws: the effect of screw type, screw orientation and imaging parameters. J Hand Surg Br 27: 165-170. doi:10.1054/jhsb.2001.0717. PubMed: 12027494. 12027494 10.1054/jhsb.2001.0717

[38] Yue Z, Zhou J, Wang X, Gui Z, Li L (2003) Preparation and magnetic properties of titanium-substituted LiZn ferrites via a sol-gel auto-combustion process. J Eur Ceram Soc 23: 189-193. doi:10.1016/S0955-2219(02)00082-1.

[39] Eggers G, Rieker M, Kress B, Fiebach J, Dickhaus H et al. (2005) Artefacts in magnetic resonance imaging caused by dental material. Magma 18: 103-111. doi:10.1007/s10334-005-0101-0. PubMed: 15785943. 15785943 10.1007/s10334-005-0101-0

[40] Muranaka H, Horiguchi T, Ueda Y, Tanki N (2011) Evaluation of RF heating due to various implants during MR procedures. Magn Reson. Med Sci 10: 11-19. 10.2463/mrms.10.11 21441723

[41] Harris ED Jr., McCroskery PA (1974) The influence of temperature and fibril stability on degradation of cartilage collagen by rheumatoid synovial collagenase. N Engl J Med 290: 1-6. doi:10.1056/NEJM197401032900101. PubMed: 4357162. 4357162 10.1056/NEJM197401032900101

[42] Yarmolenko PS, Moon EJ, Landon C, Manzoor A, Hochman DW et al. (2011) Thresholds for thermal damage to normal tissues: an update. Int J Hyperthermia 27: 320-343. doi:10.3109/02656736.2010.534527. PubMed: 21591897. 21591897 10.3109/02656736.2010.534527 PMC3609720

[43] Sapareto SA, Dewey WC (1984) Thermal dose determination in cancer therapy. Int J Radiat Oncol Biol Phys 10: 787-800. doi:10.1016/0360-3016(84)90379-1. PubMed: 6547421. 6547421 10.1016/0360-3016(84)90379-1

[44] Dewhirst MW, Viglianti BL, Lora-Michiels M, Hanson M, Hoopes PJ (2003) Basic principles of thermal dosimetry and thermal thresholds for tissue damage from hyperthermia. Int J Hyperthermia 19: 267-294. doi:10.1080/0265673031000119006. PubMed: 12745972. 12745972 10.1080/0265673031000119006

[45] Meshorer A, Prionas SD, Fajardo LF, Meyer JL, Hahn GM et al. (1983) The effects of hyperthermia on normal mesenchymal tissues. Application of a histologic grading system. Arch Pathol Lab Med 107: 328-334. PubMed: 6687797. 6687797

[46] Zderic V, Foley J, Luo W, Vaezy S (2008) Prevention of post-focal thermal damage by formation of bubbles at the focus during high intensity focused ultrasound therapy. Med Phys 35: 4292-4299. doi:10.1118/1.2975149. PubMed: 18975674. 18975674 10.1118/1.2975149 PMC2673593

[47] Sokka SD, King R, Hynynen K (2003) MRI-guided gas bubble enhanced ultrasound heating in in vivo rabbit thigh. Phys Med Biol 48: 223-241. doi:10.1088/0031-9155/48/2/306. PubMed: 12587906. 12587906 10.1088/0031-9155/48/2/306

[48] Mougenot C, Quesson B, de Senneville BD, de Oliveira PL, Sprinkhuizen S et al. (2009) Three-dimensional spatial and temporal temperature control with MR thermometry-guided focused ultrasound (MRgHIFU). Magn Reson Med 61: 603-614. doi:10.1002/mrm.21887. PubMed: 19097249. 19097249 10.1002/mrm.21887

[49] Kaasik A, Kuum M, Joubert F, Wilding J, Ventura-Clapier R et al. (2010) Mitochondria as a source of mechanical signals in cardiomyocytes. Cardiovasc Res 87: 83-91. doi:10.1093/cvr/cvq039. PubMed: 20124402. 20124402 10.1093/cvr/cvq039

[50] Ginzburg EL, Mashanskiĭ VF, Krylenkov VA, Tret’iakov AV (1976) Correlation of mitochondrial adenosine triphosphatase activity with their size following exposure of cells to higher temperatures. Tsitologiia 18: 600-604. PubMed: 133481. 133481

[51] Funk RH, Nagel F, Wonka F, Krinke HE, Gölfert F et al. (1999) Effects of heat shock on the functional morphology of cell organelles observed by video-enhanced microscopy. Anat Rec 255: 458-464. doi:10.1002/(SICI)1097-0185(19990801)255:4. PubMed: 10409818. 10409818 10.1002/(SICI)1097-0185(19990801)255:4<458::AID-AR11>3.0.CO;2-U

[52] Ji Z, Ma Y, Li W, Li X, Zhao G et al. (2012) The healing process of intracorporeally and in situ devitalized distal femur by microwave in a dog model and its mechanical properties in vitro. PLOS ONE 7: e30505. doi:10.1371/journal.pone.0030505. PubMed: 22276207. 22276207 10.1371/journal.pone.0030505 PMC3262834

[53] Liebergall M, Abu-Sneineh CH, Eylon S, Mendelson S, Segal D et al. (2000) Effect of microwave oven induced mild hyperthermia on bone viability and strength. Clin Orthop Relat Res 372: 272–279. doi:10.1097/00003086-200003000-00030. PubMed: 10738437. 10.1097/00003086-200003000-00030 10738437

[54] Welgus HG, Jeffrey JJ, Eisen AZ (1981) Human skin fibroblast collagenase. Assessment of activation energy and deuterium isotope effect with collagenous substrates. J Biol Chem 256: 9516-9521. PubMed: 6270090. 6270090

[55] Kennedy WF, Roberts CG, Zuege RC, Dicus WT (1993) Use of pulsed electromagnetic fields in treatment of loosened cemented hip prostheses. A double-blind trial. Clin Orthop Relat Res: 198-205. 8425345

[56] Olchowik G, Gaweda R, Blacha J (1992) [The influence of microwave monochromatic radiation on bone fracture union in rabbits]. Chir Narzadow Ruchu Ortop Pol 57: 297-300. PubMed: 7555283. 7555283

[57] Lehmann JF, Dundore DE, Esselman PC, Nelp WB (1983) Microwave diathermy: effects on experimental muscle hematoma resolution. Arch Phys Med Rehabil 64: 127–129. PubMed: 6830422. 6830422

[58] Seyedin SM, Segarini PR, Rosen DM, Thompson AY, Bentz H et al. (1987) Cartilage-inducing factor-B is a unique protein structurally and functionally related to transforming growth factor-beta. J Biol Chem 262: 1946-1949. PubMed: 3469199. 3469199

[59] Hauschka PV, Mavrakos AE, Iafrati MD, Doleman SE, Klagsbrun M (1986) Growth factors in bone matrix. Isolation of multiple types by affinity chromatography on heparin-Sepharose. J Biol Chem 261: 12665-12674. PubMed: 3745206. 3745206

[60] Eriksson AR, Albrektsson T (1983) Temperature threshold levels for heat-induced bone tissue injury: a vital-microscopic study in the rabbit. J Prosthet Dent 50: 101-107. doi:10.1016/0022-3913(83)90174-9. PubMed: 6576145. 6576145 10.1016/0022-3913(83)90174-9

