# Supplementary figures and images for: Effects of Low-Dose Microwave on Healing of Fractures with Titanium Alloy Internal Fixation: An Experimental Study in a Rabbit Model
Source: PLoS One. 2013 Sep 26;8(9):e75756. doi: 10.1371/journal.pone.0075756 (PMC3784417; doi:10.1371/journal.pone.0075756)

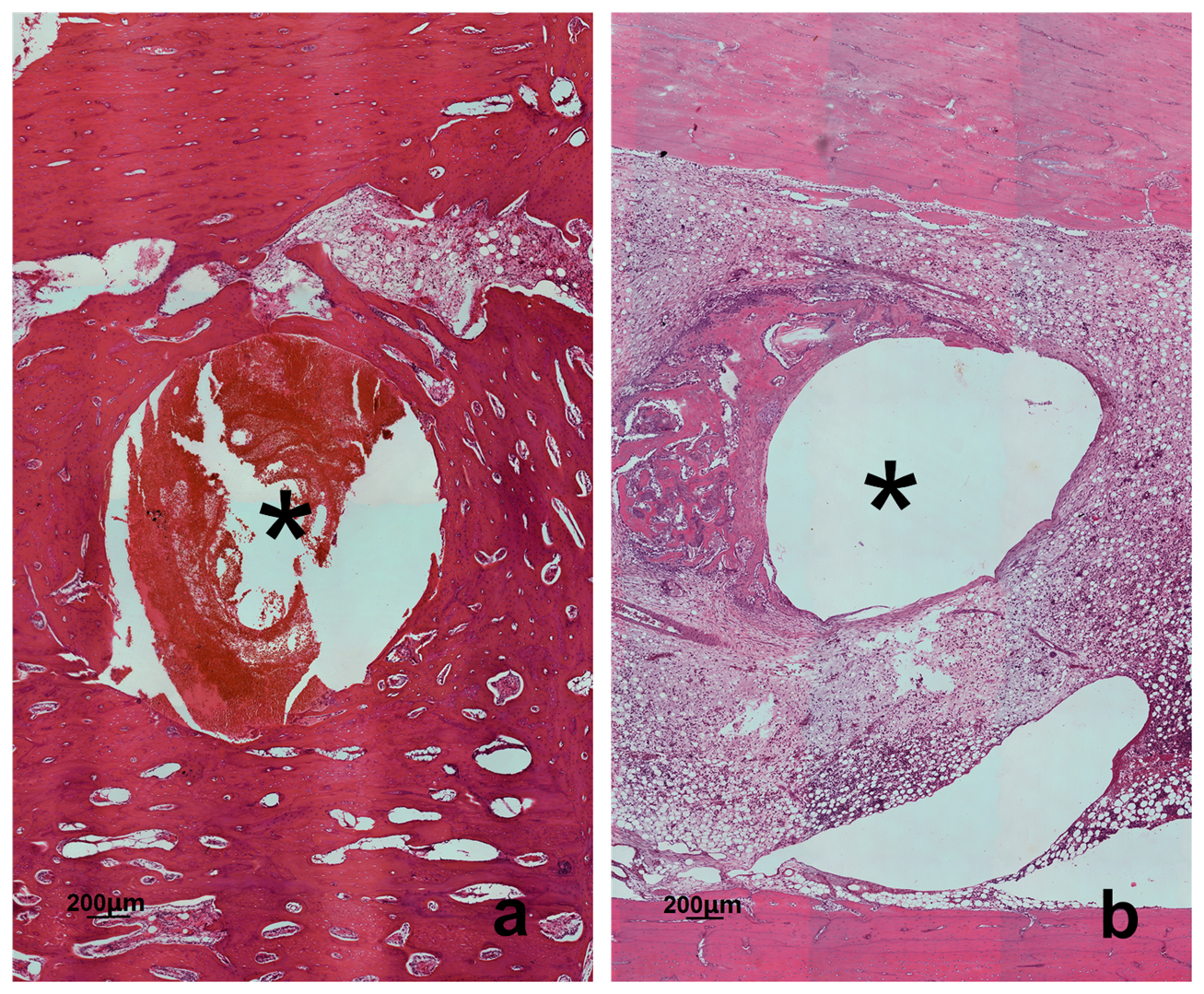

Supplement: Figure S1 — Histological manifestation of the treated femur adjacent to the screw after 30-day microwave treatment. No morphologically discernible tissue injury was observed in cortical bone (a) or bone marrow (b) of the targeted bone segment. Asterisk: the location of implanted screw. Scale bars: 200µm. (TIF) [file pone.0075756.s001.tif]
